# Mechanisms of Resistance and Therapeutic Perspectives in Immunotherapy for Advanced Head and Neck Cancers

**DOI:** 10.3390/cancers16040703

**Published:** 2024-02-07

**Authors:** Andrew Meci, Neerav Goyal, Guy Slonimsky

**Affiliations:** 1 The Pennsylvania State University College of Medicine, Hershey, PA 17033, USA; ameci@pennstatehealth.psu.edu; 2 Department of Otolaryngology-Head and Neck Surgery, Penn State Health, Milton S. Hershey Medical Center, 500 University Dr, Hershey, PA 17033, USA; ngoyal1@pennstatehealth.psu.edu

**Keywords:** immunotherapy, resistance, recurrent and metastatic head and neck squamous cell carcinoma, HNSCC, PD-1 checkpoint inhibitors

## Abstract

**Simple Summary:**

The programmed death-1 receptor monoclonal antibody treatments pembrolizumab and nivolumab have been successful in the treatment of recurrent or metastatic head and neck cancer. However, about 60% of patients will experience recurrence after this immunotherapeutic treatment. This is believed to be due to a combination of innate and adaptive tumor characteristics that suppress the body’s immune response to recurrent or metastatic head and neck cancer. This review details the mechanisms of these innate and adaptive characteristics and describes potential treatments that could be used to target and overcome these immunotherapy resistance mechanisms. These include the combinations of novel and existing therapies aimed to overcome current challenges with immunotherapy resistance and hopefully leading to improved patient outcomes.

**Abstract:**

Immunotherapy is emerging as an effective treatment for advanced head and neck cancers and interest in this treatment modality has led to rapid expansion of this research. Pembrolizumab and nivolumab, monoclonal antibodies directed against the programmed cell death-1 (PD-1) receptor, are US Food and Drug Administration (FDA)- and European Medical Agency (EMA)-approved immunotherapies for head and neck squamous cell carcinoma (HNSCC). Resistance to immunotherapy is common, with about 60% of patients with recurrent or metastatic HNSCC not responding to immunotherapy and only 20–30% of patients without disease progression in the long term. Overcoming resistance to immunotherapy is therefore essential for augmenting the effectiveness of immunotherapy in HNSCC. This review details the innate and adaptive mechanisms by which head and neck cancers can become resistant to immunotherapeutic agents, biomarkers that can be used for immunotherapy patient selection, as well as other factors of the tumor microenvironment correlated with therapeutic response and prognosis. Numerous combinations and novel immunotherapies are currently being trialed, based on better understood immune evasion mechanisms. These potential treatments hold the promise of overcoming resistance to immunotherapy in head and neck cancers.

## 1. Introduction

Head and neck cancer represents the seventh most common type of cancer worldwide, with around 660,000 diagnoses and 325,000 deaths annually [1,2]. Global incidence appears to be increasing as well, with a predicted 30% increase by 2030 [1,2]. In the USA and Europe, increasing incidence rates are primarily driven by human papillomavirus (HPV)-associated oropharyngeal cancer [3,4]. Many patients present with advanced disease, and while locoregionally confined head and neck squamous cell carcinoma (HNSCC) is principally managed with surgery, radiation, and chemotherapy, immunotherapy is gaining popularity for recurrent, persistent, locally advanced and metastatic disease [2].

A high proportion of HNSCC patients present with specific immunosuppressive traits that make for potential targets for immunotherapeutic treatment. Such traits include intrinsic baseline properties of the tumor and its microenvironment that persist despite the effects of immunotherapy. The tumor and its microenvironment are composed of a network of tumor cells, stromal cells (such as carcinoma-associated fibroblasts), endothelial cells and tumor-infiltrating immune cells including CD4^+^ and CD8^+^ T cells, regulatory T cells (Tregs), tumor-associated macrophages, natural killer (NK) cells, and myeloid-derived suppressor cells (MDSC), in addition to soluble factors like chemokines, cytokines, and growth factors like vascular endothelial growth factor (VEGF) [2,5,6,7]. HNSCC tumors have different levels of infiltration by immune cells based on their location, HPV status, and contributory risk factors (e.g., smoking) which affect their prognoses.

Advanced-stage cancers tend to exhibit upregulation of programmed death receptor-1 (PD-1), decreasing the cytotoxic effects of CD8^+^ T cells [2]. Growth factors and PD-1 are the targets of current immunotherapeutic agents proven to be effective against HNSCC. These agents include pembrolizumab and nivolumab, which target the programmed death receptor-1 pathway and are approved for treatment with or after platinum-containing chemotherapy [8,9]. While treatments all show improved survival and response especially for advanced-stage HNSCC compared to chemotherapy, resistance remains a persistent issue in HNSCC treatment with immunotherapeutic agents.

Under the current standard of care, 10–20% of early stage HNSCC and 50% of locally advanced HNSCC eventually recurs [10]. Resistance, specifically to immunotherapy, occurs in about 60% of patients, with only 20–30% of treated patients achieving long-term disease control [11,12,13]. Resistance and recurrence are associated primarily with specific tumor genetics, risk factors (smoking and HPV status), PD-L1 (programmed death ligand-1) expression, and the tumor microenvironment [6].

The purpose of this review is to summarize the current understanding of resistance to available immunotherapeutic agents approved for the treatment of advanced HNSCC along with the combinations and novel treatments being developed to overcome cancer resistance.

## 2. Immune Resistance to Standard HNSCC Treatment

Standard therapy for early-stage HNSCC includes surgery and/or primary or adjuvant radiotherapy, with or without chemotherapy, depending on clinical and pathological indications, disease stage, resectability, and distant metastasis [14]. Despite stable control of HNSCC for some patients, up to 65% of treated patients develop recurrent or metastatic disease [15].

Surgery functions to combat HNSCC through the physical removal of the bulk of diseased tissue while leaving the native immune system with a more manageable amount of cancer cells to eliminate [16]. However, surgery and perioperative interventions like anesthesia, conversely, lead to an acute immunosuppressive response [16,17]. While less impactful than the chronic immunosuppression induced by the tumor itself, surgical stress, anesthesia, and perioperative pain lead to adrenocorticoid release that in turn leads to the temporary suppression of T and natural killer cell functions [16,17]. Immunosuppression is further augmented by the cytokine tumor growth factor (TGF)-β, interleukin-1, and interleukin-6 as well as growth factors such as epidermal growth factor (EGF) and VEGF, which are released in response to tissue manipulation and the process of wound healing. In addition to immunosuppression, these growth factors could potentially drive the proliferation of residual malignant tissue following resection [16,17]. The resection of local lymphoid tissue in head and neck cancer treatment has also been proposed to be a mechanism by which immune response could be dampened following surgery [17].

The long- and short-term adverse effects of radiation therapy are well known. Radiation, even with the advent of intensity-modulated radiation therapy, can select for enhanced antigenicity of residual tumor cells in addition to inducing apoptosis in susceptible cancerous cells [17]. Immunologically, radiotherapy used to treat HNSCC has been associated with the acute systemic repression of T cell activity [18].

Platinum-based chemotherapy, which interrupts DNA crosslinking and induces apoptosis in rapidly dividing cells, has been regarded as an effective treatment for HNSCC since the late 1970s [19,20,21]. Chemotherapy has been proposed to augment immune-stimulatory activity by increasing tumor mutational burden, depleting immunosuppressive Tregs and myeloid-derived suppressor cells, normalizing neovasculature, upregulating HLA class I expression, inducing cancer cell death, and increasing cancer cell sensitivity to pro-inflammatory IFN-γ [22,23]. These agents have been shown to have a complicated pro- and anti-tumor net effect that non-specifically results in the destruction of rapidly proliferating susceptible cancerous tissue, while competitively selecting tumor cells with genetic and immunologic adaptations to evade chemotherapeutic destruction [17].

## 3. Current Immunotherapy in the Treatment of Recurrent or Metastatic HNSCC

Programmed death receptor-1 (PD-1) is expressed on the surface of activated B and T cells, Tregs, and NK cells. These are a part of the CD28 family and interact with programmed death ligands 1 and 2 (PD-L1 and PD-L2) on tumor cells and antigen-presenting cells, which are present in larger quantities in the context of HNSCC [6,24]. Under normal physiologic circumstances, the interaction between PD-1 and PD-L1/2 prevents the overstimulation of T cells and autoimmunity against native tissues [25]. However, in the tumor microenvironment, this interaction leads to inhibition of the anti-tumor immune response. This T cell suppression is primarily accomplished through interfering with T cell receptor signaling between activated T cells and regulatory T cells, enhancement of the expression of other immune checkpoint inhibitors, and interference with T cell glucose uptake [24,26,27]. In tumor-associated macrophages, increased PD-1 expression leads to the inhibition of phagocytosis and thus a reduced downstream innate and adaptive immune response [28].

Nivolumab and pembrolizumab are IgG4 monoclonal antibody anti-PD-1 checkpoint inhibitors. By blocking the interaction of PD-1 with its ligands, the checkpoint is inhibited which leads to the restoration of the anti-tumor immune response [25]. This is achieved by reversing the effects of PD-1 checkpoint overactivation, allowing for primarily CD8^+^ T cells to react immunologically to the abnormal tumor cells, as well as allowing for the antigen-presenting cell phagocytosis of tumor cells.

Following successful randomized control trials demonstrating efficacy against non-small cell lung cancer, nivolumab was applied to HNSCC in the CheckMate 141 phase III trial [29,30,31]. This trial demonstrated an overall survival benefit for nivolumab in HNSCC patients with recurrence or progression of the tumor within six months of platinum therapy compared to docetaxel, methotrexate, or cetuximab standard of care chemo and immunotherapies [31]. Within the same cohort, there was an overall survival benefit and a consistent safety profile over an extended two-year follow-up period [32]. However, this randomized trial showed limited progression-free survival and an overall response rate (ORR) of only 13.3% in the nivolumab treatment group compared to 5.8% in the standard of care treatment group [31]. Nivolumab was also shown to delay the deleterious symptoms of HNSCC, which also extended patients’ quality of life [33]. The results of the CheckMate 141 phase III trial led to approvals from the US Food and Drug Agency (FDA) and the European Medicines Agency (EMA) in 2016 and 2017 for nivolumab for recurrent and metastatic HNSCC.

Pembrolizumab was first approved by the FDA 2016 (by accelerated approval) and in 2019 (final approval) for the first-line treatment of HNSCC in patients with recurrent or metastatic disease following a phase III randomized controlled trial called KEYNOTE-048 [34]. The EMA approved this treatment for the same indications in 2019. KEYNOTE-048, like the trial of nivolumab, found that pembrolizumab improved overall survival when compared to cetuximab with chemotherapy (standard of care). However, a difference in progression-free survival was not found in the initial study period and ORRs were equal among study groups at 36% [34]. At the 4-year follow-up, the overall survival benefit of pembrolizumab was upheld as being superior to standard treatments [35]. Among included participants, pembrolizumab did not result in a decrease in quality of life compared to standard treatments [36].

In addition to the already approved pembrolizumab and nivolumab, durvalumab, atezolizumab, and avelumab are all additional PD-1 checkpoint inhibitors being evaluated for safety and efficacy at different stages of clinical trials for HNSCC. These are PD-L1 antibodies, binding to the programmed death ligand directly rather than to the receptor (PD-1), as is the mechanism of action of pembrolizumab and nivolumab.

Durvalumab is a humanized anti-PD-L1 monoclonal antibody. It has been evaluated in several high-profile phase II and III trials but, while it has been proven to be safe, it has yet to show improvement in progression-free survival when compared to standard of care treatment (platinum, 5-FU, and cetuximab—the EXTREME regimen) [37,38]. Atezolizumab is a monoclonal PD-L1 antibody currently being evaluated in a phase III trial for safety and efficacy as a surgical adjuvant against placebo for patients with recurrent or metastatic HNSCC [39]. Avelumab is a fully human monoclonal anti-PD-L1 antibody that has been evaluated in the JAVELIN phase III trial as a potential combination treatment with standard of care chemoradiation therapy and did not demonstrate improved progression-free survival [40].

## 4. Carcinogenesis of HNSCC and Immune Escape Mechanisms

At its most simple, head and neck squamous cell carcinoma involves interference with the normal process of apoptosis that epithelial cells undergo when deleterious mutations occur [41]. HNSCC also interferes with immune recognition and elimination of transformed, more specifically, neoplastic cells [42,43]. Immunotherapy through immune checkpoint blockade functionally works to counteract immunosuppressive mechanisms induced through carcinogenesis and tumor proliferation. However, this requires immune function to resume when immunotherapy targets a specific immunosuppressive driver. Thus, the failure of checkpoint blockade by immunotherapy is the result of pervasive immunosuppression caused by either intrinsic or adaptive cancer resistance mechanisms that lead to persistent tumor growth, invasion, migration, and metastasis.

### 4.1. Intrinsic Mechanisms to Anti-PD-1 Immunotherapy (Table 1, Figure 1)

HPV status divides oropharyngeal squamous cell carcinoma (OPSCC) into two distinct subgroups. HPV-positive OPSCC tends to affect younger and nonsmoking patients [44] and contrasts with HPV-negative disease, which affects older patients and is more associated with the consumption of tobacco products and alcohol and portends a worse prognosis [2,45]. The HPV-positive OPSCC microenvironment demonstrates increased immune activation and increased infiltration by T cells, T regulatory cells, and natural killer cells [44,45]. Lower immune infiltration in HPV-negative tumors contributes to an immunosuppressive environment that generally leads to a poorer prognosis [44].

**Figure 1:**
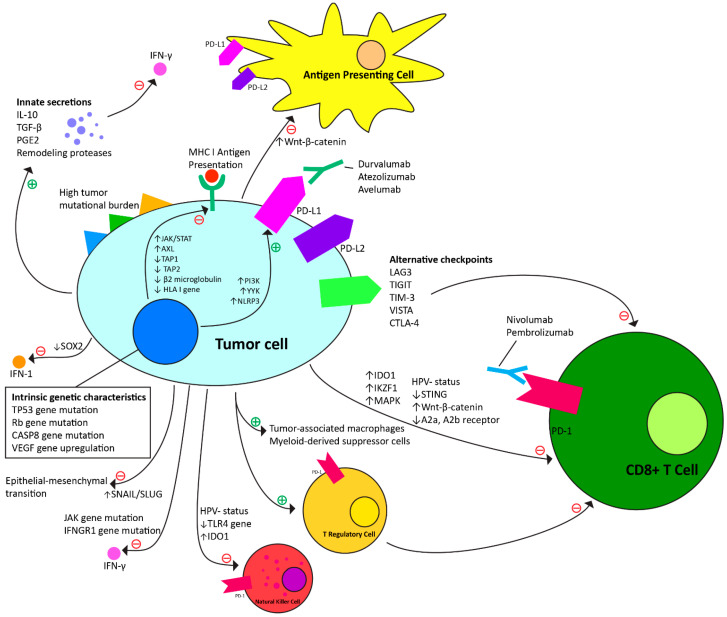
Intrinsic and adaptive resistance mechanisms to anti-PD-1 immunotherapy.

Specific genetic mutations also contribute to cancer progression. *JAK* and *IFNGR1* gene mutations result in the loss of cell sensitivity to the pro-inflammatory cytokine, interferon gamma (IFN-γ) [46,47]. There is also suppression of the stimulator of interferon genes (*STING*) pathway via the increased expression of *KDM5B*, which results in reduced CD8^+^ T cell immune response to the tumor [48]. Procaspase-8 mutation also contributes to tumor cell progeny success via constitutional binding of procaspase-8 to the FAS-associated death domain (FADD) signaling complex, inhibiting the normal process of apoptosis [49]. Toll-like receptor 4 (TLR4) expression protects HNSCC tumor cells from NK cell-induced immune attack through the antiapoptotic properties of activated NF-κB [50]. Tumor cells may also have mutations in the *TP53* and retinoblastoma genes, resulting in G1/S checkpoint dysregulation, which fosters cell proliferation despite mutations that would normally result in apoptosis [51].

Tumor-specific antigens further drive tumor progression. These neoantigens present multiple targets for T cells and increase T cell activation and recruitment. The mutational burden of tumor cells leads to increased tumor cell destruction, but also allows for the selection and proliferation of tumor cells that T cells attack ineffectively or that evade the immune response entirely. This is thought to be related to modifications to the major histocompatibility complex (MHC) antigen-presenting system, in which CD8^+^-MHC class I immunologic interaction drives selection pressure for loss of HLA class 1 loci in dominant tumor cell progenies [52,53,54,55]. This principle of immunoediting results in a temporary stable tumor state of equilibrium before the immune-evasive progeny proliferates and progresses.

HNSCC tumor cells also directly produce factors that work both locally and systemically to inhibit immune response. In addition to stimulating vascularization and recruiting endothelial cells, vascular endothelial growth factor (VEGF) inhibits dendritic cells (antigen-presenting cells), subsequently inhibiting T cell response. Interleukin-10 (IL-10), tumor growth factor beta (TGF-β), and prostaglandin E2 (PGE2) are also secreted and suppress inflammatory IFN-γ response [17]. Hypoxia drives the increased expression of hypoxia-inducible factor 1-alpha, which also stimulates VEGF expression and upregulates tumor cell expression of glucose transporters, which allows the tumor cells to convert to glycolysis-based energy production [51]. Tumor-associated macrophages secrete IL-10, TGF-β, VEGF, and remodeling proteases [56,57]. Myeloid-derived suppressor cells induce CD8^+^ cytotoxic T cell dysfunction through the production of TGF-β and local L-arginine starvation [58]. T regulatory cells also secrete IL-10 and TGF-β, but also express cytotoxic T-lymphocyte antigen-4, which downregulates the T cell immune response. T regulatory cells also release increased amounts of PD-L1, which when paired with tumor cell upregulation of PD-L1 results in decreased inflammatory cytokine release and the induction of T cell anergy and apoptosis [24].

Metastasis is dependent on tumor epithelial cell adaptations that occur prior to, during, and after detachment from the basement membrane. The key element of metastasis is the epithelial–mesenchymal transition (EMT), which is the conversion of tumor cells from epithelial to mesenchymal phenotypes [2]. HNSCC metastasis and EMT are driven by the specific regulation of transcription factors, hypoxic conditions, acquisition of stem cell properties (including activation of tumor cell signaling pathways), growth factors, and cytokines [2].

**Table 1:** Intrinsic and genetic resistance mechanisms to anti-PD-1 immunotherapy.

Characteristic of Head and Neck Cancer	Tumor Immunotherapy Resistance Mechanism
**Genetic mutations and regulatory changes**	
HPV-negative status	Lower tumor infiltration by T cells, Tregs, and NK cells [2,44,45].
*TP53*, *Rb* gene mutations	G1/S cell cycle dysregulation interfering with normal apoptosis signaling [51].
*JAK* gene mutation	Decreased sensitivity to pro-inflammatory IFN-γ [46].
*IFNGR1* gene mutation	Decreased sensitivity to pro-inflammatory IFN-γ [47].
*STING* suppression	Reduced CD8^+^ T cells in tumor microenvironment [48].
*CASP8* gene mutation	Procaspase-8 mutation to constitutionally bind to FADD, blocking normal apoptosis signaling [49].
*TLR4* gene suppression	Protects from NK cell immune attack via activated NF-κB [50].
*VEGF* gene upregulation	Contributes to hypoxic environment, driving increased expression of hypoxia-inducible factor 1-alpha, which further drives VEGF expression and glycolysis-based energy production [51].
Presence of epithelial–mesenchymal transition	Increases propensity of metastasis via regulators including transcription factors, hypoxic conditions, acquisition of stem cell properties, growth factors, and cytokines [2].
**Tumor neoantigens**	
High tumor mutational burden	Contributes to both immune stimulation and suppression. Stimulation via increase in the number of antigens the immune system has available for stimulation. Inhibitory due to mutations to the HLA I antigen presentation pathway, leading to reduced CD8^+^ T cell activation [52,53,54,55].
**Soluble tumor secretions**	
IL-10	Decreased sensitivity to pro-inflammatory IFN-γ [17].
TGF-β	Decreased sensitivity to pro-inflammatory IFN-γ [17].
PGE2	Decreased sensitivity to pro-inflammatory IFN-γ [17].
Remodeling proteases	Contribute to hypoxic environment and EMT; can contribute to a physical extracellular matrix that protects the tumor [56,57].
**Upregulated cell lines**	
Tumor-associated macrophages	Secrete IL-10, TGF-β, VEGF, and remodeling proteases [56,57].
Myeloid-derived suppressor cells	Induce CD8^+^ T cell dysfunction via production of TGF-β and local L-arginine starvation [58].
T regulatory cells	Downregulate T cell immune response via IL-10, TGF-β, and T-lymphocyte antigen-4 release; Induce T cell anergy and apoptosis; release PD-L1 [24].

### 4.2. Adaptive Resistance Mechanisms to Anti-PD-1 Checkpoint Immunotherapy (Table 2, Figure 1)

Adaptations in oncogenic signaling through the upregulation of pathways such as Wnt-β-catenin and PI3K may allow HNSCC tumors to evade PD-1 blockade. In mouse melanoma, studies have suggested that activation of the Wnt-β-catenin oncogenic pathway contributed to the impairment of dendritic cell recruitment and antigen presentation to T cells as well as loss of T cell gene expression overall [59,60]. Activation of the PI3K pathway in response to immunotherapy promotes the increased expression of CCL2 (chemokine ligand 2) and VEGF immunosuppressive cytokines and reduction in CD8^+^ T cell tumor penetration [61]. The YY1 transcription factor can also upregulate the PI3K pathway, increasing PD-L1 expression and contributing to PD-1 blockade resistance [62,63]. YY1-driven upregulation of PD-L1 may also contribute to resistance through the production of variant ligands that may act as decoys for PD-L1 antibodies in immunotherapeutic treatment, allowing for the PD-1/PD-L1 checkpoint to remain intact despite targeted therapy [64]. The NLRP3 pathway also contributes to PD-L1 upregulation via the recruitment of granulocytic myeloid-derived suppressor cells and subsequent immunosuppression [65].

The JAK/STAT pathway has been implicated in innate and adaptive loss of IFN-γ receptors on tumor cells as well as deficits in antigen-presenting components, which contributes to the loss of inflammatory immune responses negating the effectiveness of immunotherapy [66,67,68]. Other studies have found that the SOX2 inhibition of the STING gene can lead to immune resistance via blockade of the IFN-1 pathway in HNSCC [69]. Antigen presentation deficits have also been tied to adaptive mutations in TAP1, TAP2, β2-microglobulin, HLA class I, and AXL receptor tyrosine kinase genes, which contribute to the downregulation of MHC class I antigen-presenting machinery and prevention of T cell activation [52,70,71,72].

The upregulation of lymphocyte activation gene-3 (*LAG3*), T cell immunoglobulin and ITIM (TIGIT)/CD155, T cell immunoglobulin mucin-3 (TIM-3), V domain-containing Ig suppressor of T cell activation (VISTA), and cytotoxic T lymphocyte antigen (CTLA-4) pathways represent alternative immune checkpoints that are all modified in response to the blockade of PD-1 [12]. *LAG3* upregulation, like PD-1, normally moderates immune reaction and prevents autoimmunity but in the context of malignancy contributes to impaired T cell proliferation and cytokine production [73]. Similar mechanisms of action and resistance have been proposed for the TIGIT, TIM-3, VISTA, CTLA-4 pathways [74,75,76,77]. TIM-3 and CTLA-4 upregulation has also been implicated in T cell exhaustion, a state of deficient effector function and the consequent immune evasion of HNSCC cells [71,76,78].

Adenosine and ATP have also been studied in relation to immunosuppression in the tumor microenvironment and tumor resistance to anti-checkpoint-based immunotherapy. While ATP promotes immune response, adenosine contrarily leads to immune cell suppression through binding to A2a and A2b receptors present on T cells, NK cells, neutrophils, dendritic cells, and macrophages. In the context of resistance to immunotherapy, tumor cells develop upregulated related CD28 and adenosine pathways which in turn leads to the suppression of CD8^+^ T cell response [79].

Increased expression of SNAIL and SLUG transcription factors contribute to the increased expression of transforming growth factor-β (TGF-β) and matrix metalloproteinases, which has been associated with epithelial–mesenchymal transition and metastasis despite PD-1 blockade [80,81]. Tumor cells can also upregulate and directly secrete the immunosuppressive factor indoleamine 2,3-dioxygenase-1 (IDO1) which increases expression of arginase-1 by tumor cells, causing degradation of L-arginine needed for NK and T cell survival and proliferation [82,83].

Recruitment of immunosuppressive cells has also been tied to resistance to the immunotherapeutic treatment of cancer. Upregulation of Tregs has been proposed as contributing to the increased recruitment and infiltration of myeloid-derived suppressor cells, which inhibit the proliferation and function of T cells through the nitric oxide pathway [84]. The IKZF1 transcription factor also contributes to immune infiltrate recruitment [85]. MAPK pathway activation induces VGEF and IL-8 release to inhibit T cell recruitment and has also been implicated in the cross-activation of other oncogenic pathways such as PI3K and JAK/STAT [86].

Finally, external modification of the native microbiome with antibiotic use has also been proposed as a resistance mechanism, though the underlying mechanisms of this resistance have yet to be entirely delineated [87].

**Table 2:** Adaptive resistance mechanisms to anti-PD-1 immunotherapy.

Adaptive Modification Pathway	Immunotherapy Resistance Mechanism
**Oncogenic signaling**	
Wnt-β-catenin upregulation	Suppresses dendritic cell recruitment and antigen presentation to T cells; decreases T cell gene expression [59,60].
PI3K upregulation	Increases expression of CCL2 and VEGF immunosuppressive cytokines; reduces CD8^+^ T cell tumor penetration; increased extrinsic PD-L1 expression [61].
YY1 upregulation	Upregulates PI3K; increases extrinsic PD-L1 and variant “decoy” ligand expression [62,63,64].
**Alternative checkpoints**	
*LAG3* upregulation	Contributes to impaired T cell proliferation and cytokine production [73].
TIGIT upregulation	Contributes to impaired T cell proliferation and cytokine production [75,76].
TIM-3 upregulation	Contributes to impaired T cell proliferation and cytokine production; contributes to T cell exhaustion [71,75].
VISTA upregulation	Contributes to impaired T cell proliferation and cytokine production [74,77].
CTLA-4 upregulation	Contributes to impaired T cell proliferation, exhaustion, and cytokine production [71,76].
**Alternative immunosuppression**	
NLRP3 upregulation	Upregulates PD-L1 expression via the recruitment of granulocytic myeloid-derived suppressor cells [65].
JAK/STAT upregulation	Contributes to IFN-γ receptor loss on tumor cells and deficits in antigen presentation [66,67,68].
SOX2 downregulation	Inhibits STING gene and contributes to blockade of the IFN-1 inflammatory pathway [69].
TAP1 downregulation	Contributes to suppression of antigen presentation machinery [70].
TAP2 downregulation	Contributes to suppression of antigen presentation machinery [70].
β2-microglobulin downregulation	Contributes to suppression of antigen presentation machinery [70,71].
HLA I gene downregulation	Reduced MHC I antigen-presenting machinery leading to decreased CD8^+^ T cell activation [52].
AXL upregulation	Contributes to suppression of antigen presentation machinery [72].
A2a and A2b receptor upregulation	Contributes to suppression of CD8^+^ T cell immune response [79].
SNAIL/SLUG upregulation	Increases expression of TGF-β and matrix metalloproteinases, which are associated with EMT [80,81].
IDO1 upregulation	Reduces proliferation of T and NK cells through expression of arginase-1 by tumor cells (degrades L-arginine needed for cell survival) [82,83].
IKZF1 upregulation	Inhibition of immune infiltrate recruitment [85].
MAPK upregulation	Induces VGEF and IL-8 release inhibiting T cell recruitment; cross-activates JAK/STAT and PI3K [86].
Treg upregulation	Increased recruitment of myeloid-derived suppressor cells, which immunosuppress via the nitric oxide pathway [84].
Antibiotic therapy	Modifies the native microbiome and likely the immune microenvironment as a result [87].

### 4.3. Predictors of Treatment Response

The American Society of Clinical Oncology (ASCO) gives evidence-based recommendations for immunotherapy in the treatment of recurrent or metastatic HNSCC. Locoregionally advanced disease, a common presentation of HNSCC, is typically first treated with multimodality treatments including surgery, radiation, and/or chemotherapy like cisplatin (most commonly). Advanced recurrent or metastatic disease is treated systemically, and treatments can include chemotherapy and/or immunotherapy [88].

The ASCO recommends that patients with recurrent or metastatic HNSCC undergo PD-L1 immunohistochemistry testing (evidence quality high, strong recommendation) and that tumor mutational burden (TMB) testing should be performed when immunohistochemistry is not available or if the patient has a rare tumor type (evidence quality high, strong recommendation). PD-L1 combined positive score (CPS) or a high TMB results both correlate with clinical benefit to PD-1 inhibitors, according to available clinical evidence. ASCO strongly recommends pembrolizumab monotherapy or pembrolizumab, platinum, and fluorouracil as first-line agents for CPS scores above the positive threshold (≥ 1), pembrolizumab, platinum, and fluorouracil for patients with CPS < 1, and pembrolizumab or nivolumab for patients with platinum-refractory or metastatic HNSCC regardless of CPS status. Additionally, there are weak recommendations to offer PD-1 inhibitors to patients with recurrent or metastatic nasopharyngeal cancer, patients with recurrent or rare head and neck cancers with high TMB, and patients with PD-L1-positive recurrent or metastatic salivary gland cancer [88].

Studies have shown that high levels of PD-L1 expression are associated with higher response rates to immunotherapeutic treatment as well as improved outcomes compared to those with different tumor characteristics [25,89]. However, it should be noted that PD-L1 levels are not standardized, and PD-L1-negative cancers have also shown responses to anti-PD-1 checkpoint inhibitor immunotherapy [89]. For this reason, alternative methods for measuring programmed death checkpoint targets have been proposed. Measurement of PD-L2 has been proposed based on the finding that the molecule has a stronger affinity to PD-1 receptors and that this may be a greater driver of immunosuppression than PD-L1 in the programmed death checkpoint [90,91,92]. This is supported by the finding that PD-L2 expression independently predicts response to anti-PD-1 immunotherapy even in the setting of PD-L1 negativity (PD-L2 is present in >60% of PD-L1-negative tumors) [93]. Other studies have suggested the use of combined positive score (CPS), the sum of PD-L1-positive tumor cells, lymphocytes, and macrophages divided by total viable tumor cells, multiplied by 100. Several trials have found an increased ORR associated with a CPS greater than 1 [6,12,34]. Positron emission tomography and magnetic resonance imaging have been studied as potential PD-L1 expression predictors as well [90].

TMB and microsatellite instability (MSI), its underlying genetic process, ultimately lead to neoantigen formation through somatic mutations [6]. Higher TMB has been associated with improved patient outcomes when treated with anti-PD-1 immunotherapy [25,94,95]. This has led to the FDA approving nivolumab and pembrolizumab treatment for cancer of any histology that is positive for microsatellite instability [96] (it is important to note that HNSCC has a low incidence of MSI). The FDA has also approved pembrolizumab for the treatment of a wide variety of recurrent solid tumors with high TMB, defined as greater than or equal to 10 mutations/megabase [6].

The immune microenvironment of recurrent or metastatic HNSCC has also been cited as a potential indicator of response to immunotherapy. Given that increased CD8^+^ T cell infiltration has been associated with an improved response to anti-PD-1 immunotherapy agents in other forms of cancer, gene expression profiling and the interferon gamma gene expression signature assay have been developed to measure the degree of T cell activation [25,97,98,99]. Additionally, HPV-negative status, which is associated with lower immune infiltration, is associated with a reduced response to checkpoint inhibitor immunotherapy [44].

As discussed previously, resistance to immunotherapy occurs in approximately 60% of patients with recurrent or metastatic HNSCC. Given this statistic, clinicians need to select suitable patients for immunotherapeutic treatment and appropriately counsel patients regarding prognosis when undergoing such therapy. Prognostic indicators for anti-PD-1 checkpoint inhibitors include checkpoint targets, tumor genomics and neoantigens, tumor immune microenvironment, tumor causative factors, and radiologic features.

## 5. Overcoming Resistance to Immunotherapy (Table 3)

High rates of innate and adaptive resistance to PD-1 blockade necessitate the further development of therapies targeted to this challenging patient population. Several mechanisms have been proposed to combat anti-PD-1 resistance and improve patient outcomes.

Biomarker usage for patient selection and prognosis prediction is currently extremely limited. The ASCO currently only recommends the measurement of PD-L1 using IHC and tumor mutational burden in certain circumstances [88]. Given the complexities of anti-PD-1 resistance, the biomarker profile of patients should be expanded to better understand each individual oncological profile. This expanded biomarker profile could include measurements of PD-L2 and CPS, which have also been associated with improved response to PD-1 checkpoint inhibitors by previous studies [6,12,34,93]. Further inclusion of tumor and patient genetic profiles, especially those involved in immune resistance mechanisms, would potentially contribute to better targeted and individualized immunotherapy.

Research into new treatment options beyond pembrolizumab or nivolumab monotherapies and pembrolizumab combined with platinum and fluorouracil chemotherapies is being pursued in a plethora of Phase I, II, and III clinical trials. These new potential treatments for advanced head and neck cancer include new applications of anti-PD-1 monoclonal antibodies (described in Section 3), alternative checkpoint targets, combination strategies for immunotherapies and other treatments, oncolytic virus therapies, therapeutic cancer vaccines, and adoptive cell therapies.

### 5.1. Combination Strategies

#### 5.1.1. Salvage Surgery

Adjuvant PD-1 blockade immunotherapy in combination with salvage surgery is being evaluated in multiple clinical trials as a means of improving the overall survival of patients with recurrent or metastatic HNSCC [100,101]. Surgery in this context functions as previously described by reducing tumor bulk and leaving a lesser amount of cancerous tissue for the immune system to combat. The adjuvant PD-1 blockade in this context then promotes the removal of immunosuppressive barriers to immune response against residual cancer cells. A recent phase II clinical trial has shown a tolerable safety profile and improved disease-free survival compared to historical control samples at two-year follow-up [101].

#### 5.1.2. Chemotherapy

First-line chemotherapy, while often effective, has also been associated with PD-L1 expression, thus supporting the addition of PD-1 blockade treatment in treatment-resistant cases [102]. Combinations of PD-1 inhibitors with 5-fluorouracil (5-FU), cisplatin, and/or paclitaxel are all underway [22]. Of note, the KEYNOTE-048 phase III study has already demonstrated that there was an improvement in overall survival, but not progression-free survival, for patients receiving platinum, 5-FU, and pembrolizumab compared to the EXTREME regimen, especially for patients who were PD-L1-positive. The ASCO already recommends this regimen as first-line treatment for recurrent or metastatic HNSCC [34,88,103].

#### 5.1.3. Radiation Therapy

Radiotherapy in combination with PD-1 blockade has been shown to reverse T cell exhaustion and propagate oligoclonal T cell expansion as well as resulting in anti-tumor activity through a non-redundant mechanism in animal models [104,105]. Despite these theories and efficacy in animal models, the Phase II and III GORTEC and JAVELIN Head and Neck 100 trials have failed to demonstrate improved progression-free survival [22,104]. Nonetheless, there are several additional ongoing trials such as KEYSTROKE and REPORT, both of which are examining the potentially synergistic effects of radiation combined with PD-1 checkpoint inhibition [104].

#### 5.1.4. Combinations with Other Immune Checkpoint Inhibitors

Alternative immunosuppressive checkpoints are important mechanisms by which recurrent and metastatic HNSCC resists PD-1 immunotherapy. Consequently, blockade of these checkpoints has become an area of interest for potential new treatment combinations with PD-1, especially due to their non-redundant nature. These checkpoints include CTLA-4, *LAG3*, TIGIT, and TIM-3.

CTLA-4 is expressed on the surface of CD8^+^ T cells and competitively (with the stimulatory CD28 receptor) binds to the B7 ligand, which when bound suppresses antigen presentation via MHC proteins. Ipilimumab is an anti-IgG1 anti-CTLA-4 monoclonal antibody checkpoint blockade treatment that has been evaluated with the anti-PD-1 agents durvalumab and nivolumab. Following a demonstratable synergy in the treatment of metastatic melanoma [106], HNSCC treatment regiments including durvalumab have not shown positive survival results in the phase II and III trials called CONDOR and EAGLE [107,108]. Results from the CheckMate 651 phase III trial combining nivolumab and ipilimumab did not show meaningful improvement in progression-free survival despite a favorable safety profile when compared with the EXTREME standard of care regimen [39].

LAG3 is an immunosuppressive checkpoint pathway that primarily suppresses T cells through recognition of the MHC class II molecule. Relatlimab, a LAG3 checkpoint inhibitor, has already been approved by the FDA for the treatment of advanced melanoma and multiple phase I and II clinical trials are underway to examine the survival benefits of LAG3-inhibitors both as monotherapy and in combination with PD-1 inhibitors within the context of HNSCC and other solid tumors [25,109].

Multiple studies examining TIGIT blockade with the monoclonal antibody tiragolumab are underway in the context of HNSCC and other solid tumors [110]. Of particular note is the SKYCRAPER-09 trial examining tiragolumab in combination with atezolizumab compared to placebo in a phase II trial for recurrent and metastatic HNSCC. This follows the phase II CITYSCAPE trial that showed an improved objective response rate (ORR) and safety profile of this treatment combination when compared to atezolizumab alone [111].

The TIM-3 checkpoint causes immunosuppression by reducing production of cytokines and by inducing apoptosis of T cells [112]. Early studies of TSR-022 (cobolimab) and MGB453, two monoclonal antibodies to TIM-3, are underway in the context of anti-PD-1 combinations and solid tumors [102,113].

#### 5.1.5. Combinations with Other Immune-Stimulating Molecules

Cetuximab, an FDA-approved monoclonal antibody for HNSCC, inhibits EGFR signal transduction, which promotes antigen presentation and immune response to the tumor cells. Given its non-redundant mechanism of action to PD-1 checkpoint inhibitors, cetuximab combination therapies with anti PD-1 treatment have been widely studied. A recent systematic review and meta-analysis of seven phase I, II, and III trials has demonstrated that combination therapy results in a significantly improved ORRs and one-year overall survival when compared to anti-PD-1 monotherapy in HPV-negative recurrent or metastatic HNSCC. This effect, however, was not seen with HPV-positive disease [114].

In combination with PD-1 inhibitors, danvatirsen (AZD9150, a STAT3 inhibitor) has been shown to be safe by the SCORES study for patient use and suggested anti-tumor activity and further trials are underway [25,115,116].

CXCR2, a cytokine receptor associated with IL-8, has also been shown to be overexpressed in HNSCC. The inhibitor for this receptor, AZD5069, did not improve ORR and had a high rate of adverse events when tested in combination with durvalumab in the SCORES study [117].

Epacadostat, an IDO1 inhibitor, has been tested in combination with PD-1 inhibitors in advanced solid tumors (the ECHO-304/KEYNOTE-669 study) and patients were found to have a tolerable safety profile with a relatively high ORR [118]. However, a subsequent phase III study called CheckMate 9NA/ECHO-310 that examined the same treatment combination was halted prematurely due to negative results in a melanoma phase III treatment trial [119]. Another phase III trial, ECHO-304/KEYNOTE-669, is still ongoing and is examining the progression-free survival benefit of epacadostat with pembrolizumab [120]. Navoximod, another IDO1 inhibitor therapy, has also been combined with atezolizumab in a phase I trial for patients with solid tumors, demonstrating an adequate safety profile and indeterminant results on efficacy [121].

NKG2A is expressed on CD8^+^ T cells as well as NK cells and contributes to immunosuppression in the tumor microenvironment. The monalizumab blockade of NKG2A in combination with anti-PD-1 treatment has been the subject of several recent studies. The UPSTREAM phase II trial comparing monalizumab combination therapy with durvalumab with standard of care protocols for recurrent or metastatic HNSCC has thus far not demonstrated improvements to progression-free survival, though final results are pending [122].

B7H3 is found on cytotoxic and helper T cells and it has been posited to negatively regulate T cell function [123]. B7H3 blockade with retifanlimab and enoblituzumab with anti-PD-1 therapy has been studied in a phase I study demonstrating a favorable ORR and has led to the development of a phase II/III study demonstrating thus far an acceptable safety profile and anti-tumor activity results [124].

Inducible co-stimulator of T cells agonist (ICOS) is an immunosuppressive stimulator that upregulates Tregs, though it also has been shown to have anti-tumor immune action when the same pathway is activated in cytotoxic CD8^+^ T cells [125,126]. Despite these findings and findings of a synergy between PD-1 checkpoint inhibitors and ICOS agonists, both the INDUCE-3 and INDUCE-4 phase II/III trials have been terminated prematurely due to the insufficient efficacy of feladilimab, the ICOS agonist employed in these studies [127].

Lenvatinib is a multikinase inhibitor of VEGF which was the subject of a previous phase II trial; it was demonstrated that a combination of lenvatinib with pembrolizumab led to an improved ORR. However, a subsequent phase III trial involving patients with recurrent or advanced HNSCC was terminated early due to failure to achieve significant improvement in the overall survival of enrollees [128,129].

#### 5.1.6. Oncolytic Viral Immunotherapy

Oncolytic viral therapy can be used in combination with immune checkpoint inhibitors to enhance the response rates of patients with recurrent or metastatic HNSCC. This is accomplished by several proposed mechanisms including direct oncolysis, systemic anti-tumor immunity, and destruction of tumor vasculature. Direct oncolysis occurs as the result of viral invasion and proliferation within the tumor that leads to the eventual destruction of the host cell and infection of subsequent adjacent tumor cells while the body mounts an immune response. Anti-tumoral immunity is accomplished in this setting both through innate mechanisms with dendritic and NK cell inflammatory activation and through adaptive mechanisms involving CD4^+^ and CD8^+^ responses following the release of tumor-associated antigens from infected and lysed tumor cells. Tumor cell lysis and release of contents also result in vascular endothelial cell damage and death [130].

In combination with anti-PD-1 therapy, co-administration of oncolytic viruses can overcome innate and adaptive immunosuppressive mechanisms that lead to failure of immune checkpoint inhibition. Adenovirus and herpes simplex virus (HSV) have been most extensively studied, but vaccinia virus, vesicular stomatitis virus, and the measles virus are also in clinical trials for head and neck cancer treatment [130,131].

### 5.2. Other Treatments in Development

#### 5.2.1. Cancer Vaccines

Cancer vaccines represent a potential alternative or adjuvant option to immune checkpoint inhibitor immunotherapy for HPV+ HNSCC. Therapeutic vaccines (which are distinct from prophylactic HPV vaccines) are designed to induce antigen-specific, cell-mediated cytotoxicity that targets specific tumor antigens [132,133]. The most effectively studied antigens within HNSCC thus far are the melanoma antigen-encoding gene (MAGE), HPV-E6, E7, Epstein–Barr virus-related latent membrane protein-2, MUC-1, Wilm’s tumor-1, survivin, carcinoembryonic antigen, and epidermal growth factor receptor [134,135].

Alternatively, therapeutic vaccines by a different design could selectively target elements of the immunosuppressive tumor microenvironment, which, similar to previously discussed immune checkpoint inhibitors, would rely on normal native immune functioning after immunosuppressive elements are inhibited [132]. Given this approach, a combination treatment with checkpoint inhibitors is also being evaluated in trials [136,137].

Over 40 major trials related to therapeutic vaccines are underway for HNSCC, all of which are in various points within Phase I and II and many of which are being used in combination with immune checkpoint inhibitor agents [132]. These vaccines function via a variety of delivery platforms including autologous tumor cell vaccines, allogenic whole tumor vaccines, dendritic cell vaccines, peptide vaccines, DNA vaccines, RNA vaccines, and viral vaccines [132,133]. Of note, mRNA vaccines promise to overcome immune tolerance through neoantigen peptide targeting and several trials are underway examining their use as mono- and combination therapy with PD-1-inhibitors. mRNA vaccines can also code for antibodies, presenting a novel delivery mechanism [133].

#### 5.2.2. Adoptive Cellular Therapy

Adoptive cellular therapy has been shown to have great anti-tumor efficacy in hematologic malignancies and is currently studied in the context of HNSCC. This therapy genetically re-engineers native cells to target tumor-specific antigens and release pro-inflammatory cytokines to eliminate these tumor cells. This, in effect, engineers a hostile immune environment against the tumor using adoptive immunity and in opposition to the local immunosuppressive tumor microenvironment.

Common targets of chimeric antigen T (CAR-T) cell therapy in hematologic malignancies have included EGFR and HER2 [138]. Despite success in treating other malignancies, the translation of CAR-T adoptive immune therapy to HNSCC has been unsuccessful due to a concerning safety profile. In the context of HNSCC, EGFR targeting with CAR-T causes gastrointestinal, respiratory, hematological, and immunological toxicity due to the ubiquity of EGFR in normal tissues. However, efforts have been made to increase CAR-T efficacy and safety by directing delivery locally to the surgical site and by targeting proteins overexpressed in HNSCC tumor tissue compared to local adjacent tissue, namely FAP, HER3, and NKGD2. Several Phase I and II clinical trials are underway to examine the safety profiles and utilities of CAR-T cell therapy directed against these protein targets [138].

Engineered T cell receptor T (TCR-T) therapy utilizes T cell receptor isolation and peptide/HLA engineering to recognize a wide array of intracellular tumor-associated antigens. TCR-T can overcome resistance to checkpoint blockade because T cells are specifically designed and delivered rather than relying on functional native T cells for immune destruction of the tumor following alleviation of immune suppression [139]. Given this theoretical framework, there is great potential for using TCR-T as a future mono- or combined HNSCC therapy.

Natural killer cell therapy uses a similar model to CAR-T cell therapy, though applies the same principles to natural killer cells. By tailoring NK cells to tumor-specific antigens, they provide an alternative immune attack mechanism to T cells. There have also been fewer instances of graft vs. host disease reported with NK therapy due to their greater transplantability. In vivo trials have shown similar efficacy to CAR-T therapy and clinical trials for advanced solid tumors are ongoing [140].

**Table 3:** Prospective treatments to overcome current resistance to checkpoint inhibiting immunotherapy.

Treatment Modality	Mechanism of Action in Overcoming Resistance	Simplified Results of Clinical Trials
**Additional anti-PD-1 antibodies**		
Durvalumab	Humanized PD-L1 monoclonal antibody.	Monotherapy is safe but has not shown progression-free survival improvement compared to standard of care [37,38].
Atezolizumab	PD-L1 monoclonal antibody.	Under investigation as adjuvant treatment to surgery. Results pending [39].
Avelumab	Fully human monoclonal anti-PD-1 monoclonal antibody.	No progression-free survival benefit compared to placebo in combination with standard of care [40].
**Anti-PD-1 combination with standard of care treatments**		
Salvage surgery	Reduction of tumor bulk	Phase II trials have shown safety and improved disease-free survival compared to historical samples [101].
Chemotherapy	Increases TMB; depletes Tregs and MDSCs; normalizes neovasculature; upregulates HLA I; induces cancer cell death; increases sensitivity to IFN-γ.	Phase III trial has shown improvement in overall survival with platinum, 5-FU, and pembrolizumab compared to the EXTREME regimen [34].
Radiotherapy	Reverses T cell exhaustion; propagates oligoclonal T cell expansion; direct anti-tumor activity (in animal models).	Past trials have not shown progression-free survival benefits. Several phase III trials are ongoing [22,104].
**Anti-PD-1 combination with non-redundant immune checkpoint inhibitors**		
CTLA-4 inhibitors (Ipilimumab)	Blocks binding of CTLA-4 to B7 ligand, which restores antigen presentation via MHC proteins.	Multiple phase III trials have shown no improvement in overall survival when combined with durvalumab or nivolumab [39,107,108].
LAG3 inhibitors (Relalimab)	Blockade restores MHC II function.	Phase I and II trials are underway. Results pending [25,109].
TIGIT inhibitors (Tiragolumab)	Blockade restores T cell immune function against tumor cells.	A phase II trial is underway in combination with atezolizumab compared to placebo. Another phase II trial showed improved ORR compared to atezolizumab alone [110,111].
TIM-3 inhibitors (Cobolimab, MGB453)	Blockade restores production of cytokines and prevents apoptosis of T cells.	Early trials are underway for two different monoclonal antibody blockades of TIM-3. Results pending [102,113].
**Anti-PD-1 combinations with other immune stimulating molecules**		
EGFR inhibitors (cetuximab)	Inhibition of EGFR promotes antigen presentation an immune response to tumor cells.	Combinations with PD-1 inhibitors have shown improved ORR and overall survival in HPV-related disease [114].
STAT3 inhibitors (AZD9150)	Blockade inhibits immunosuppressive transcription factor.	Phase I studies have shown a tolerable safety profile and suggested anti-tumor activity [115,116].
CXCR2 inhibitors (AZD5069)	Blockade of pro-inflammatory cytokine receptor (IL-8 predominantly).	Tested with durvalumab, the combination did not improve patient ORR and had a high rate of adverse events [117].
IDO1 inhibitors (Epacadostat, Navoximod)	Blockade decreases arginase-1 expression and restores T and NK cell proliferation.	Epacadostat and pembrolizumab have been shown to be safe but did not show positive results in a melanoma phase III trial [119,120]. Navoximod with atezolizumab is in Phase I testing for solid tumors [121].
NKG2A inhibitors (Monalizumab)	Blockade restores CD8^+^ T and NK cell function.	Monalizumab with durvalumab with standard of care treatment has not shown improvements to progression-free survival. Phase II trial results are pending [122].
B7H3 Inhibitors (Enoblituzumab)	Blockade restores CD8^+^ T cell function.	Combination with retifanlimab (PD-1 inhibitor) has shown an improved ORR. A phase II/III study has so far shown acceptable safety and anti-tumor activity results [124].
ICOS inhibitors (Feladilimab)	Blocks Treg upregulation.	Phase II/III trials have not shown survival benefit [127].
VEGF inhibitors (Lenvatinib)	Inhibition deters hypoxic environment.	A phase III trial combining lenvatinib with pembrolizumab did not show survival benefit [128,129].
**Anti-PD-1 combinations with oncolytic virus immunotherapy**		
Adenovirus	Stimulate direct oncolysis, systemic anti-tumor immunity, and destruction of tumor vasculature.	Phase 1 studies involving different virus variants and PD-1 inhibitor combinations are underway. Results pending [130,131].
Herpes simplex virus		
**Cancer vaccines**		
Cell-mediated cytotoxicity vaccines	Induces antigen-specific, cell-medicated cytotoxicity that targets specific tumor antigens.	Over 40 major trials related to therapeutic vaccines are underway for HNSCC, all of which are in various points within Phase I and II phases and many of which are being used in combination with PD-1 checkpoint inhibitors [132].
Direct targeting of immunosuppressive elements	Selectively target and inhibit elements of the immunosuppressive tumor microenvironment.	
**Adoptive cellular therapy**		
CAR-T cell therapy	Genetically re-engineered native T cells target tumor-specific antigens and release pro-inflammatory cytokines.	EGFR targeting with CAR-T causes gastrointestinal, respiratory, and hematological toxicity. New strategies that target local adjacent tumor tissue antigens like FAP, HER3, and NKGD2 are in phase I and II clinical trials to assess safety [138].
Natural killer cell therapy	Genetically re-engineered autologous NK cells target tumor-specific antigens.	In vivo trials have shown efficacy and phase 0, I, and II are underway [140].
Engineered T cell receptor T therapy	T cell receptor isolation and peptide/HLA engineering to recognize intracellular tumor-associated antigens.	No trials yet underway for HNSCC [139].

## 6. Conclusions and Future Directions

Despite promising results for current immunotherapy with anti-PD-1 immune checkpoint inhibitors for selected patients with persistent, recurrent, or metastatic head and neck squamous cell carcinoma, high rates of treatment failure still occur, mainly through the ability of cancer cells to evade the immune system. It is now understood that this immune evasion is both innate and adaptive in nature, and eventually enables tumor growth, local invasion, and metastasis through inhibition of the native immune system. PD-1 inhibitors can often disable this immune suppression through the PD-1 checkpoint pathway; however, in many cases, modifications of cancer cells and/or the microenvironment ultimately suppress the immune response to cancerous tissue.

The growing understanding of the mechanisms of advanced head and neck cancer resistance to immunotherapy is essential for the detection of novel biomarkers that will enable the molecular profiling of patients and cancer cells. This would help to determine the potential effectiveness of immunotherapy derived from the overall propensity for treatment resistance. Furthermore, new treatment targets, combinations, and modalities are under development to address these, now better understood, elements of tumor-driven immunosuppression. These trials will hopefully lead to advancements in the effectiveness of immunotherapy and improved outcomes of patients with advanced HNSCC.
